# Residual Peripheral Blood CD26^+^ Leukemic Stem Cells in Chronic Myeloid Leukemia Patients During TKI Therapy and During Treatment-Free Remission

**DOI:** 10.3389/fonc.2018.00194

**Published:** 2018-05-30

**Authors:** Monica Bocchia, Anna Sicuranza, Elisabetta Abruzzese, Alessandra Iurlo, Santina Sirianni, Antonella Gozzini, Sara Galimberti, Lara Aprile, Bruno Martino, Patrizia Pregno, Federica Sorà, Giulia Alunni, Carmen Fava, Fausto Castagnetti, Luca Puccetti, Massimo Breccia, Daniele Cattaneo, Marzia Defina, Olga Mulas, Claudia Baratè, Giovanni Caocci, Simona Sica, Alessandro Gozzetti, Luigiana Luciano, Monica Crugnola, Mario Annunziata, Mario Tiribelli, Paola Pacelli, Ilaria Ferrigno, Emilio Usala, Nicola Sgherza, Gianantonio Rosti, Alberto Bosi, Donatella Raspadori

**Affiliations:** ^1^Hematology Unit, Department of Medicine, Surgery and Neuroscience, Azienda Ospedaliera Universitaria Senese, University of Siena, Siena, Italy; ^2^Department of Hematology, Tor Vergata University Hospital, Rome, Italy; ^3^IRCCS Ca’ Granda – Maggiore Policlinico Hospital Foundation, Milan, Italy; ^4^Department of Hematology, University of Firenze, Florence, Italy; ^5^Department of Hematology, Clinical and Experimental Medicine, University of Pisa, Pisa, Italy; ^6^Hematology Unit Bianchi Melacrino Morelli Hospital, Reggio Calabria, Italy; ^7^Hematology Division, Azienda Ospedaliera Città della Salute e della Scienza, Torino, Italy; ^8^Fondazione Policlinico Universitario A Gemelli IRCSS Università Cattolica Sacro Cuore, Rome, Italy; ^9^Azienda Ospedaliera S. Maria, Terni, Italy; ^10^Hematology Division, Ospedale Mauriziano, Torino, Italy; ^11^Department of Experimental, Diagnostic and Specialty Medicine, Institute of Hematology and Medical Oncology “L. & A. Seràgnoli”, University of Bologna, Bologna, Italy; ^12^Hematology, Biotecnologie Cellulari ed Ematologia, University “La Sapienza”, Rome, Italy; ^13^Hematology Unit, Department of Medical Sciences, University of Cagliari, Cagliari, Italy; ^14^Hematology Federico II University, Naples, Italy; ^15^Hematology Unit, Maggiore Hospital University of Parma, Parma, Italy; ^16^Hematology, Cardarelli Hospital, Naples, Italy; ^17^Division of Hematology and BMT, Department of Medical and Morphological Researches, University of Udine, Udine, Italy; ^18^Department of Medical Biotechnologies, University of Siena, Siena, Italy; ^19^Hematology Unit, Ospedale Oncologico A. Businco, Cagliari, Italy; ^20^Hematology Unit, IRCCS “Casa Sollievo della Sofferenza”, San Giovanni Rotondo, Italy

**Keywords:** chronic myeloid leukemia, CD26, leukemic stem cells, TKI, minimal residual disease, flow cytometry

## Abstract

Chronic myeloid leukemia (CML) patients in sustained “deep molecular response” may stop TKI treatment without disease recurrence; however, half of them lose molecular response shortly after TKI withdrawing. Well-defined eligibility criteria to predict a safe discontinuation up-front are still missing. Relapse is probably due to residual quiescent TKI-resistant leukemic stem cells (LSCs) supposedly transcriptionally low/silent and not easily detectable by BCR-ABL1 qRT-PCR. Bone marrow Ph+ CML CD34^+^/CD38^−^ LSCs were found to specifically co-express CD26 (dipeptidylpeptidase-IV). We explored feasibility of detecting and quantifying CD26^+^ LSCs by flow cytometry in peripheral blood (PB). Over 400 CML patients (at diagnosis and during/after therapy) entered this cross-sectional study in which CD26 expression was evaluated by a standardized multiparametric flow cytometry analysis on PB CD45^+^/CD34^+^/CD38^−^ stem cell population. All 120 CP-CML patients at diagnosis showed measurable PB CD26^+^ LSCs (median 19.20/μL, range 0.27–698.6). PB CD26^+^ LSCs were also detectable in 169/236 (71.6%) CP-CML patients in first-line TKI treatment (median 0.014 cells/μL; range 0.0012–0.66) and in 74/112 (66%), additional patients studied on treatment-free remission (TFR) (median 0.015/μL; range 0.006–0.76). Notably, no correlation between BCR-ABL/ABL^IS^ ratio and number of residual LSCs was found both in patients on or off TKIs. This is the first evidence that “circulating” CML LSCs persist in the majority of CML patients in molecular response while on TKI treatment and even after TKI discontinuation. Prospective studies evaluating the dynamics of PB CD26^+^ LSCs during TKI treatment and the role of a “stem cell response” threshold to achieve and maintain TFR are ongoing.

## Introduction

For many years since their appearance in the treatment scenario of chronic myeloid leukemia (CML), TKIs have been an extraordinary highly effective life-long treatment (1, 2). More recently, the demonstration that a fraction of CML patients in sustained “deep molecular response” (DMR) may indeed stop TKI treatment without recurrence of disease introduced the concept of “treatment-free remission” (TFR). Clinical data suggest that treatment duration in addition to deepness of response may influence TFR rate and criteria for safe TKI discontinuation include a TKI duration >3 years and a MR4 of at least 1 year (3–6). However, up to date, it is not possible to predict relapse and approximately 40–60% of DMR patients stopping TKIs will lose their response and require retreatment (7, 8) while others will maintain TFR even with detectable molecular disease in some cases (4, 5). Based on available data it is likely that relapse after TKI discontinuation may be due to the persistence of leukemic stem cells (LSCs) surviving TKIs through the activation of several BCR-ABL1-independent pathways (9–11). If this is the scenario, qRT-PCR, the most sensitive assay to monitor disease status in CML patients, may be inappropriate to quantify residual quiescent CML LSCs, which may be transcriptionally low/silent, while surviving indefinitely into tumor-specific hypoxic niches (12). In the recent past, several authors, including us, have attempted to quantify residual LSCs in bone marrow of TKI treated CML patients with discordant results mainly due to technical difficulties and relative low numbers of studied patients (13–16).

Based on these premises, the possibility to easily monitor the dynamics of CML LSCs during TKI treatment, in terms of rate and timing of reduction, could represent an alternative attempt to quantify the actual “cellular” residual disease and thus to identify optimal candidates for TKI discontinuation in addition to and may be even beyond standard molecular response.

Leukemic stem cells supposedly reside within the CD34^+^/CD38^−^/Lin^−^ fraction, which is the same compartment where also normal hematopoietic stem cells (HSCs) are found. Different biomarkers have been tested to better discriminate the leukemic clones from the normal counterpart and recently, Herrmann et al. identified CD26 (dipeptidylpeptidase IV) as a potential biomarker for the quantification and isolation of CML LSCs in BM samples of CML patients (17). In fact, in contrast to other tested antigens such as CD90 and IL-1RAP, which are co-expressed on CML LSCs, acute myeloid leukemia (AML) LSCs and normal HSCs, CD26 was the only marker, expressed in all tested CP CML patients, which was not present on CD34^+^/CD38^−^ SC in normal BM or on LSCs of other myeloid neoplasms (17, 18). In this study, the authors reported that BM sorted CD26^+^ LSCs cells were confirmed to be BCR-ABL1^+^ and in short-term colony assays and in long-term culture-initiating cells, colony cells derived from CD26^+^ LSCs (obtained from CML patients) contained BCR/ABL1mRNA, whereas cells of colonies derived from CD26^−^ SC (from the same patients) did not contain BCR-ABL1. The capability of CD34^+^/CD38^−^/CD26^+^ LSCs fraction to produce a myeloid (granulo-monocytic) BCR-ABL1^+^ engraftment was also confirmed by xenotransplantation experiment in NSG mice (17). Valent et al. clarified the functional role of CD26 and discovered that this cell surface enzyme, when present, can induce degradation of cytokine ligands such as SDF-1, a molecule that normally attracts HSCs and LSCs into the BM niche *via* CXCR4 receptor (19). This degradation facilitates LSCs mobilization from the BM niche, suggesting that CD26 could have a fundamental role in the regulation of LSCs–niche interaction and help LSCs escape TKI treatment. Finally, BM CD26^+^ LSCs appeared to decrease substantially during a successful treatment with TKIs indicating that CD26 could also be a good predictive biomarker for monitoring CML patients during therapy (20) and that flow cytometry approach could be a useful tool for the identification of CML LSCs on BM samples by using a CD45^+^/CD34^+^/CD38^−^/CD26^+^ panel as a strict gating strategy (18, 20). Recently, Warfving and colleagues revealed a great heterogeneity of BM LSCs through the combination of flow cytometry and single-cell molecular analysis; however, the crucial role of CD26 antigen was confirmed by the evidence that most insensitive TKI cells of the BM LSC compartment were defined by a specific Lin^−^CD34^+^CD38^−/low^CD45RA^−^cKIT^−^CD26^+^ phenotype (21).

Based on these evidences, considering that routine monitoring of residual LSCs from bone marrow is not practical, the present study aims were: (i) to explore the feasibility of CD26^+^LSC flow cytometry evaluation in peripheral blood (PB) in CML patients; (ii) to quantify circulating LSCs during TKI treatment and during TKI discontinuation, and (iii) to search a correlation, if any, between number of residual LSCs and molecular response (BCR-ABL/ABL1^IS^ ratio).

## Patients and Methods

### Patients Cohort

Between June 2015 and June 2017, CP CML patients either newly diagnosed or during their first-line treatment with any approved TKI, as well as patients in TFR after first line TKI discontinuation entered this not interventional cross-sectional study (including a total of 468 patients). In addition, a substantial number of patients with blood disorders other than CML and normal HSCs donors treated with granulocyte-colony stimulating factor (G-CSF) were asked to participate to the study as negative control. All subjects provided an informed consent in accordance with their institution policy.

### Flow Cytometry

**Figure 1:**
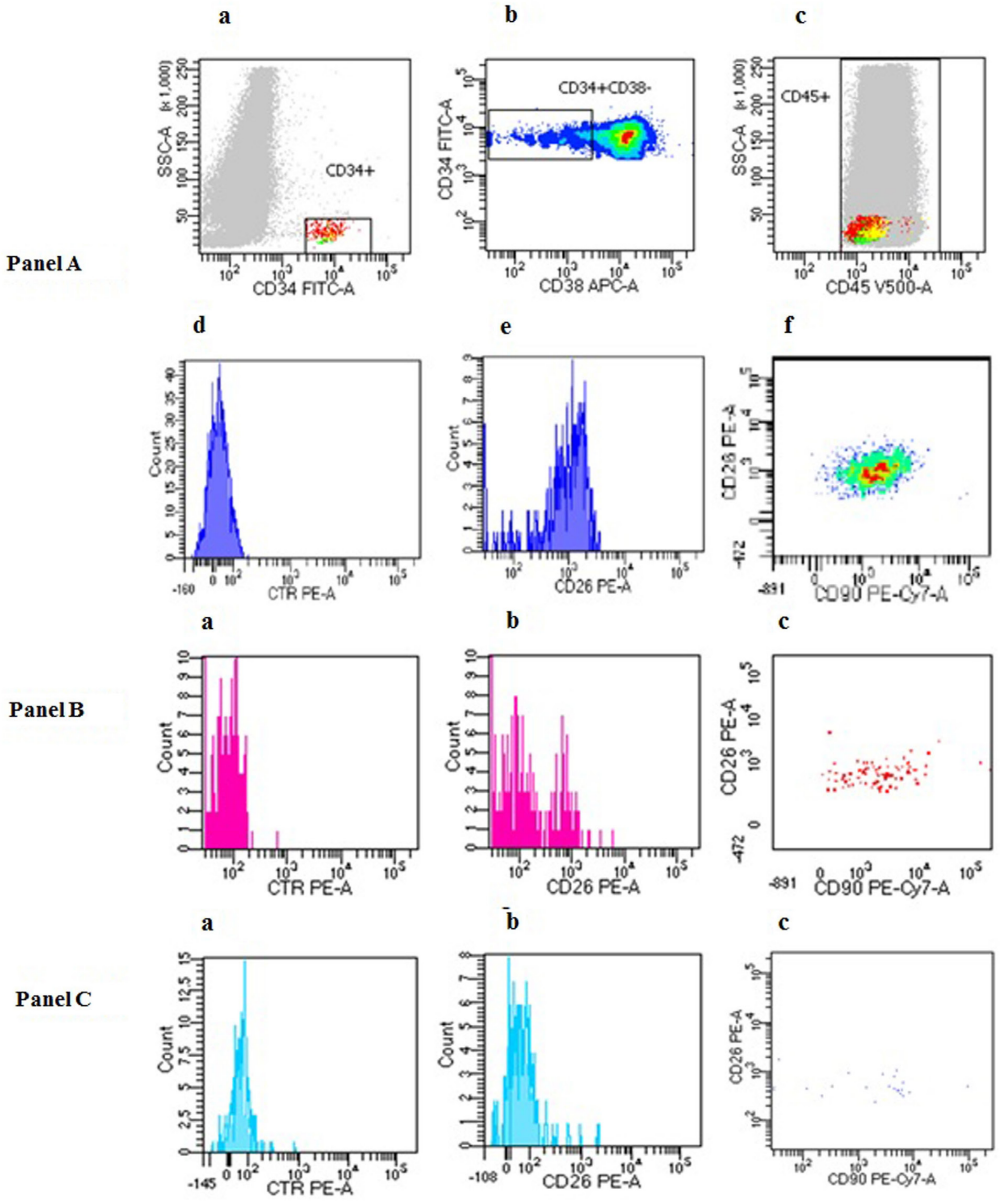
Peripheral blood (PB) CD26^+^ leukemic stem cells (LSCs) flow cytometry evaluation in chronic myeloid leukemia (CML) patients at diagnosis, during TKI treatment and during treatment-free remission. **(Panel A)** *PB CD26^+^ LSC evaluation in a representative CML patient at diagnosis*. CD34^+^CD38^−^CD26^+^ cells were identified using sequential gate with the aim to exclude debris and doublets. First, CD34 gate was performed only on viable cells identified by FSC and SSC light properties (a); then, exclusively CD34^+^CD38^−^ population was gated (b). Both CD34^+^ and CD34^+^CD38^−^ populations show CD45^dim^ expression (c). Negative control (d); CD26 expression on CD34^+^CD38^−^ population (e), and CD90 co-expression on CD34^+^CD38^−^CD26^+^ cell (f). In the case here depicted, the percentage of CD26^+^ cells within the CD34^+^CD38^−^ fraction was 93%. **(Panel B)** *PB CD26*^+^ *LSC evaluation in a representative CML patient during TKI treatment*. Circulating CML LSCs were documented in about 70% CML patients on treatment regardless of type of TKI, length of treatment, and molecular response. This flow cytometry panel shows the presence of PB residual CD26^+^ LSCs in a CML patient on imatinib for 29 months. In this case, the percentage of CD26^+^ cells within the CD34^+^CD38^−^ fraction was 8% corresponding to 0.131/μL of circulating CD26^+^ LSCs. (a) control tube; (b) test tube CD26 expression; (c) co-expression of CD90 on CD26^+^LSCs. **(Panel C)** *PB CD26*^+^ *LSC evaluation in a representative patient during treatment free remission (TFR)*. This flow cytometry panel shows the presence of PB residual CD26^+^ LSCs in a CML patient during TFR. In this case, the percentage of CD26^+^ cells within the CD34^+^CD38^−^ fraction was 1.2% corresponding to 0.0054/μL of circulating CD26^+^ LSCs. (a) control tube; (b) test tube CD26 expression; (c) co-expression of CD90 on CD26^+^LSCs.

Six milliliters of EDTA PB samples (all subjects) and 2 mL of EDTA BM samples (only CML patients at diagnosis) were collected and centrally analyzed within 24 h at Flow Cytometry lab in Siena. Preliminary experiments showed that within 24 h, cell viability was superior to 80%. In all PB or BM samples, CD26 expression was evaluated by a standardized multiparametric flow cytometry analysis on CD45^+^/CD34^+^/CD38 population using a four-color staining protocol with lyse stain wash procedure. Briefly, red cell lysis was performed with ammonium chloride (BD Biosciences), 1:10 diluted in deionized water, using Lyse Wash Assistant instrument (BD Biosciences). After lysis, 2.0 × 10^6^ leukocytes were incubated with a custom made lyophilized pre-titrated antibody mixture test tube (BD™ Lyotubes, BD) including CD45 V500 (BD Pharmingen clone 2D1), CD34 FITC (BD Pharmingen clone 581), CD38 APC (BD Pharmingen clone HIT2), and CD26 PE (BD Pharmingen clone M-A261) antibodies and a BD stain control tube lacking the CD26. To reach a sensitivity of 10^−5^, acquisition and analysis of at least 1.0 × 10^6^ cells was performed in all samples by FACSCanto II flow cytometer using a DIVA 8 software (BD, Biosciences). Additionally, to ensure reproducible results over time, we followed a standardized protocol that implied adjustments of FACS internal parameters, using the CS&T System (BD Biosciences), to keep constant the instrument performance by correcting wear of lasers and fluidic instability (22). Figure 1 Panel A, a–e shows step by step CD26^+^LSC flow cytometry analysis of one representative CML PB sample at diagnosis.

In a smaller cohort of patients, the co-expression of CD90 PeCy7 (BD Pharmingen clone 5E10) was tested to further confirm that CD45^+^CD34^+^CD38^−^CD26^+^ population belongs to the stem cell compartment (Figure 1 Panel A, f).

### Quantitative RT-PCR

Simultaneously to CD26^+^ LSCs measurement CML patients were evaluated for PB BCR-ABL1 transcript levels by standard qRT-PCR analysis, according to European Leukemia Net (ELN) criteria (1). All blood samples were tested within the Italian molecular laboratory net for CML (Labnet CML) under stringent quality control testing. Undetectable level of BCR-ABL1 is referred as 0 mRNA copies detected in 32,000 ABL copies (MR^4.5^) or in 100,000 ABL copies (MR^5^).

### Statistical Analysis

To estimate putative differences in the levels of each single biochemical variable, we employed the Mann–Whitney *U*-test and the Wilcoxon test for comparisons between and within groups if applicable. The Kendall rank correlation coefficient was used to detect putative relations among the measurable variables. Furthermore, the Mood test and bi-linear relation modeling were employed to evaluate the putative relations among parametric and the nonparametric data such as standard MR categories. *p* < 0.05 was accepted as statistically significant. All calculations were performed using the SPSS library version 13 (SPSS Inc. Chicago, IL, USA).

## Results

### CD26^+^ LSCs Evaluation at Diagnosis

During study time frame, 120 newly diagnosed CML patients were tested for circulating PB CD26^+^LSCs. In 80/120 (67%), the analysis was performed simultaneously also in BM samples. All PB and BM samples of CP-CML patients scored positive for the presence of CD26 LSCs (120/120, 100%). In particular, within the CD34^+^/CD38^−^ stem cell fraction, the median percentage of CD26^+^ cells was 21% (range 0.56–77.16) and 36.9% (range 4.2–98.8) in BM and PB samples, respectively. However, the median absolute number of CD26^+^ cells/μL [calculated as follow: (no. WBCs/μL) × (% of CD34^+^/CD38^−^/CD26^+^ on CD45^+^ cells)] was comparable in BM and PB samples: 18.38/μL (range 0.063–309.12) and 19.20/μL (range 0.27–698.6), respectively. This concordance of results allowed us to quantify LSCs directly from PB samples. Figure 1 Panel A, a–e shows CD26^+^LSC flow cytometry analysis of one representative CML PB sample at diagnosis. Of note, in CML PB samples, at diagnosis, the number of CD26^+^ LSCs correlated with the total WBC count (*p* < 0.01).

### CD26^+^ LSCs Evaluation During TKI Treatment

Peripheral blood samples of 236 CP CML patients on first-line treatment with any approved TKI were also evaluated for circulating CD26^+^ LSCs during a routinely molecular response assessment according to ELN guidelines. Type of TKI, treatment length, molecular response, and quantification of circulating LSCs are summarized in Table 1. PB CD26^+^ LSCs were detectable in 169/236 (71.6%) patients with a median number of 0.014 CD26^+^cells/μL (range 0.0012–0.66). Figure 1 Panel B, a,b shows PB CD26^+^LSC evaluation in a representative CML patient on TKI treatment (imatinib) for 29 months. PB BCR-ABL/ABL^IS^ ratio in these 169 samples with measurable CD26^+^ LSCs showed a median value of 0.007% (range 0–61). Kendall rank correlation coefficient used to analyze the relationship between the variables showed no correlation between BCR-ABL/ABL^IS^ ratio and number of residual CD26^+^ LSCs (*r* = 0.118 *p* = 0.097). Even when we compared measurable CD26^+^ LSCs and BCR-ABL log reduction (expressed in standard MR categories, i.e., MR^1^, MR^2^, MR^3^, MR^4^, MR^4.5^) according to the Mood test and bi-linear relation model, no significant correlation was found (*U* = −0.105, *p* = 0.091). In 67/236 (28.4%) patients, PB CD26^+^ LSCs were undetectable; in this sub-group, a specific analysis by the Kendall rank correlation coefficient found no correlation with the concomitant degree of molecular response (*r* = 0.14, *p* = 0.097). Similarly, in 45/236 (19%) patients with undetectable BCR-ABL1/ABL1^IS^ and 191/236 (81%) with detectable BCR-ABL1/ABL1^IS^, no correlation was found with the number of residual PB CD26^+^ LSCs [Kendall rank correlation coefficient: *r* = 0.019 (*p* = 0.09) and *r* = 0.157 (*p* = 0.094)] (Table 1). Finally, no correlation between BCR-ABL1/ABL1^IS^ and number of PB CD26^+^ LSCs was shown even considering the whole cohort of 236 patients (*r* = 0.101, *p* = 0.112).

**Table 1:** Peripheral blood CD26^+^ leukemic stem cells (LSCs) evaluation in chronic myeloid leukemia (CML) patients during TKI treatment.

	Total pts	IMATINIB treated pts	NILOTINB treated pts	DASATINIB treated pts
No.	236	108	90	38

Median TKI treatment duration (months)	36 (1–195)	68 (2–195)	24 (1–132)	12 (2–133)

CML patients with detectable peripheral blood (PB) CD26^+^ LSCs (%)^f^	169/236 (71.6%)	77/108 (71.2%)	65/90 (72.2%)	27/38 (68.4%)

Median BCR-ABL1^IS^ratio (range)^a^	0.007 (0–61)	0.006 (0–61)	0.01 (0–8.1)	0.014 (0–0.35)

Median No. CD26^+^ LSCs/μL (range)^a^	0.014 (0.0012–0.66)	0.014 (0.0029–0.24)	0.019 (0.0012–0.66)	0.0099 (0.0018–0.09)

CML patients with undetectable PB CD26^+^ LSCs (%)	67/236 (28.4%)	31/108 (28.8%)	25/90 (27.8%)	11/38 (31.6%)

Median BCR-ABL1^IS^ratio (range)^b^	0.005 (0–0.74)	0.002 (0–0.74)	0.006 (0–0.68)	0.099 (0.002–0.39)

CML patients with undetectable BCR-ABL1^ISc^	45/236 (19%)	25/108 (23.1%)	17/90 (18.8%)	3/38 (8%)

Median no. CD26^+^ LSCs/μL (range)^d^	0.0102 (0–0.0744)	0.0058 (0–0.0346)	0.022 (0–0.0744)	0.0185 (0.0133–0.0237)

CML patients with detectable BCR-ABL1^IS^	191/236 (81%)	83/108 (76.9%)	73/90 (81.2%)	35/38 (92%)

Median no. CD26^+^ LSCs/μL (range)^e^	0.0079 (0–0.66)	0.0088 (0–0.24)	0.00815 (0–0.66)	0.0018 (0–0.29)

*^a^Kendall rank correlation coefficient: r = 0.118 (p = 0.097)*.

*^b^Kendall rank correlation coefficient: r = 0.14 (p = 0.097)*.

*^c^Patients with undetectable BCR-ABL1 in a sample with a calculated detection limit of ≥4.5 logs below standardized baseline*.

*^d^Kendall rank correlation coefficient: r = 0.019 (p = 0.091)*.

*^e^Kendall rank correlation coefficient: r = 0.157 (p = 0.094)*.

*^f^Even when we compared detectable PB CD26^+^ LSCs and BCR-ABL log reduction (expressed in standard MR categories, i.e., MR^1^, MR^2^, MR^3^, MR^4^, MR^4.5^) according to the Mood test and bi-linear relation model, no significant correlation was found (U = −0.105, p = 0.091)*.

### CD26^+^ LSCs Evaluation During TFR

A total of 112 CP CML patients in TFR for a median of 31 months (range 1–152) were evaluated. All 112 patients were in first line treatment at the time of TKI withdrawal. Sixty-eight out of 112 (61%) patients discontinued imatinib treatment and were in TFR for a median of 38 months (range 3–152); 28/112 (25%) patients discontinued nilotinib and were in TFR for a median 19 months (range 1–78) and 16/112 (14%) patients were in TFR for a median of 32 months (range 9–90) after dasatinib treatment (Table 2).

**Table 2:** Peripheral blood CD26^+^ leukemic stem cells (LSCs) evaluation in chronic myeloid leukemia patients during treatment-free remission.

	Total pts	IMATINIB treated pts	NILOTINIB treated pts	DASATINIB treated pts
No.	112	68	28	16
Median treatment-free remission time (range)	31 (1–152)	38 (3–152)	19 (1–78)	32 (9–90)
No. pts with detectable CD26^+^ LSCs (%)	74/112 (66.1%)	49/68 (72%)	17/28 (61%)	9/16 (56%)
Median no. CD26^+^LSCs/μL (range)	0.015/μL (0.006–0.76)	0.02/μL (0.006–0.76)	0.016/μL (0.007–0.67)	0.016/μL (0.007–0.47)

When considering the whole cohort of TFR patients, PB CD26^+^ CML LSCs were measurable in 74/112 (66%) patients with a median number of 0.015/μL (range 0.006–0.76). Table 2 shows CD26^+^ LSCs evaluation in the whole cohort and according to previous TKI treatment: persistence of circulating CD26^+^ LSCs was found in 72, 61, and 56% of imatinib, nilotinib, and dasatinib treated patients, respectively (*p* = 0.069 among groups).

**Figure 2:**
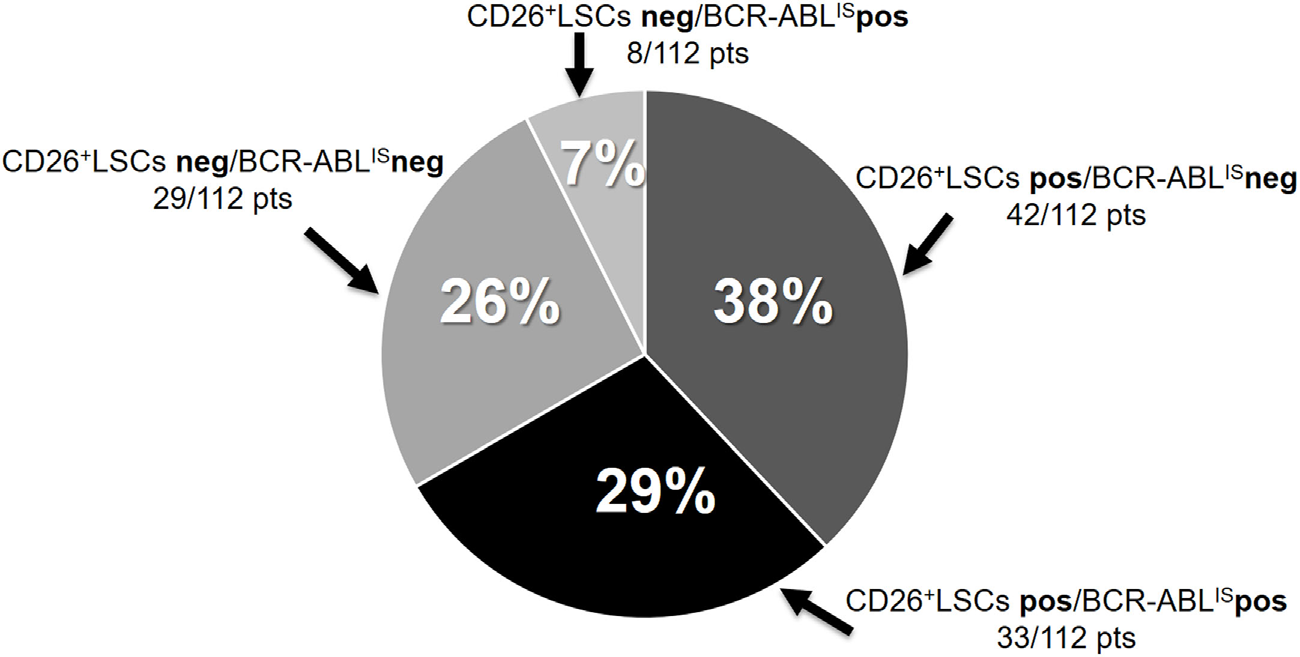
Peripheral blood CD26^+^ leukemic stem cells and BCR-ABL1^IS^ transcript in the whole cohort of treatment-free remission patients. BCR-ABL1^IS^ neg = undetectable BCR-ABL1 transcript [no BCR-ABL1 copies/32,000 ABL copies (MR^4.5^) or no BCR-ABL1 copies/100,000 ABL copies (MR^5^)].

Regarding molecular response, only 41/112 (36%) patients showed detectable BCR-ABL1 copies (median BCR-ABL1/ABL^IS^ ratio 0.0062 range 0.0032–0.493). Indeed, 33/112 (29%) patients showed both circulating CD26^+^ LSCs and detectable BCR-ABL1, 29/112 (26%) patients scored negative for both determinations, while 42/112 (38%) patients showed detectable CD26^+^ LSCs and undetectable BCR-ABL1. Only 8/112 (7%) patients showed measurable BCR-ABL1 copies and undetectable CD26^+^ LSCs (Figure 2). Kendall rank correlation coefficient, Mood test and bi-linear relation model of the whole cohort showed no correlation between BCR-ABL1/ABL^IS^ ratio and number of residual LSCs, while a significant linear inverse correlation was found between number of circulating CD26^+^ LSCs and TFR duration (*r* = −0.050 e *p* = 0.0107).

### Validation of Results

#### PB CD26^+^ LSCs Are Philadelphia-Positive

**Figure 3:**
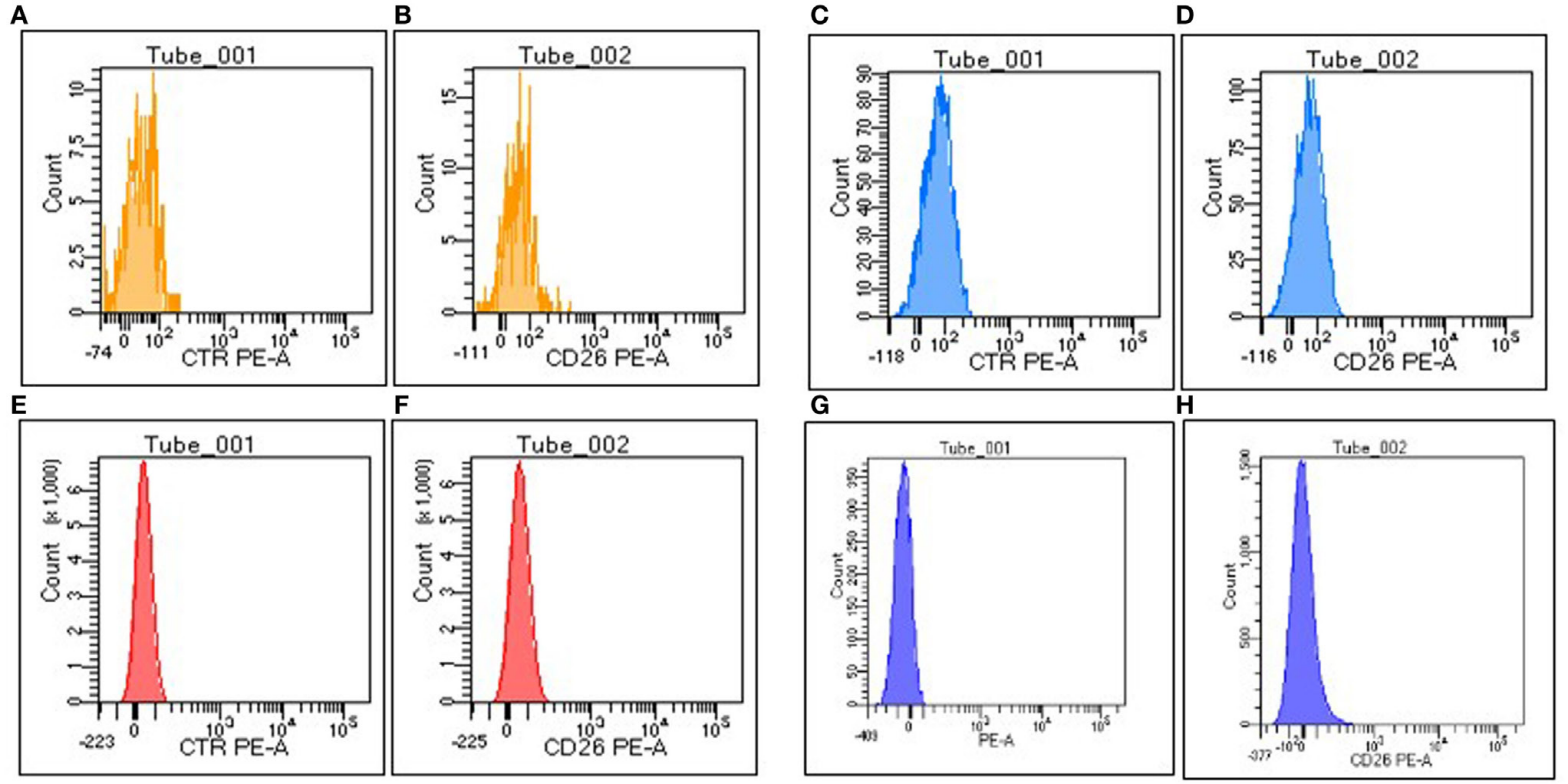
Representative histograms of the expression of isotype control and CD26 on CD34^+^CD38^−^ fraction in peripheral blood (PB) of normal subjects and patients affected by other blood disorders. **(A)** Negative control, **(B)** CD26 in a representative case of idiopathic myelofibrosis, **(C)** negative control, **(D)** CD26 in a representative case of a Ph+ acute lymphoblastic leukemia, **(E)** negative control, **(F)** CD26 in a representative case of acute myeloid leukemia, **(G)** negative control, **(H)** CD26 in a representative normal donor after receiving G-CSF.

To validate CML specificity of PB CD26^+^ LSCs detected during TKI treatment and to confirm that also circulating CD26^+^ LSCs are Philadelphia-positive, we performed dual fusion BCR-ABL1 FISH analysis of PB sorted CD34^+^/CD38^−^/CD26^+^ in five CML patients at 3–6 months after starting TKI treatment. A median of 10 cells (range 5–13) were detected in CD34^+^/CD38^−^/CD26^+^ fraction, and all cells were found BCR-ABL1 positive, confirming the results previously reported in BM experiments (17). To further confirm CML specificity of PB CD26^+^ LSCs flow cytometry assessment, we tested for the presence of CD45^+^/CD34^+/^CD38^−^/CD26^+^ cells a consistent number of PB samples from patients affected by other blood disorders, such as idiopathic myelofibrosis (IMF) (12 samples) Ph+ acute lymphoblastic leukemia (Ph+ ALL, 5 samples) AML (10 samples). In addition, nine PB samples from healthy donors undergoing HSCs mobilization after G-CSF were evaluated as well. Despite the presence of variable amounts of circulating CD45^+^/CD34^+^/CD38^−^ stem cells, in none of these PB samples studied a co-expression of CD26 was recorded [Figure 3 shows the absence of circulating CD26^+^ LSCs in a representative IMF patient (a, b), Ph+ ALL patient (c, d), AML patient (e, f), and healthy donor (g, h), respectively].

#### CD34^+^/CD38^−^/CD26^+^ Population Belongs to the Stem Cell Compartment

To confirm that CD34^+^/CD38^−^/CD26^+^ cells are indeed true “stem cells,” the concomitant expression of CD90 antigen was also evaluated. We tested PB and BM samples of 32 newly diagnosed CML patients and, in all of them, CD26^+^ LSCs fraction showed co-expression of CD90. Figure 1 shows CD26^+^ LSCs expressing CD90 in a representative patient at diagnosis (Figure 1 Panel A, e). The co-expression of CD90 was retained also in residual circulating CD26^+^ LSCs during TKI treatment (Figure 1 Panel B, c) and after TKI discontinuation (Figure 1 Panel C, c).

## Discussion

This study represents the first attempt to measure, in a large cohort of CP CML patients, blood circulating LSCs at diagnosis, during TKI treatment and even during TFR, a condition that represent the actual best surrogate for “cure” in CML patients. In our hands, PB CD26^+^LSC flow cytometry assay appeared feasible, reproducible, specific, and sensitive and thus suitable for routine monitoring.

First, we clearly demonstrated that 100% of CML patients at diagnosis show CML-specific CD26^+^ LSCs and that CD26^+^ LSCs absolute number is superimposable in PB and BM samples allowing us to easily quantify LSCs directly in PB.

Second, as somehow expected, we found that the majority of CML patients undergoing any of approved first line TKI treatment still harbored measurable residual LSCs, even when displaying a consistent DMR. No correlation with type of TKI and persistence of circulating LSCs was found. Surprisingly, the absolute number of PB CD26^+^ LSCs did not correlate with the molecular response expressed as BCR-ABL1^IS^ ratio or as MR categories and most of patients with undetectable BCR-ABL1 transcript, still showed circulating CD26^+^LSCs. These data support the evidence that qRT-PCR accurately measures transcript level but cannot as precisely estimate the actual CML cell number. Additionally, our results suggest that molecular response mainly refers to transcriptionally active CML progenitor cells while quiescent, TKI-resistant, CML LSCs may be transcriptionally low/silent and thus much less detectable by qRT-PCR. The evidence that in our study about 30% of CML patients on TKI treatment do not show detectable CD26^+^ LSCs by flow cytometry may be due to the sensitivity of the assay but may also reflect a real exhaustion of LSCs.

The third important message of our study is that, yet not unexpectedly, we have found residual circulating CD26^+^LSC also in 66% of CML patients studied while in prolonged and stable TFR. No statistically significant correlation was found between previous TKI treatment and persistence of PB LSCs even though we registered a higher percentage of patients with residual CD26^+^ LSCs in the subgroup in TFR after discontinuing imatinib. Again, no clear correlation was found between absolute number of circulating LSCs and the degree of molecular response (expressed in BCR-ABL1/ABL^IS^ ratio) and it is noteworthy that in 38% of patients with “undetectable” BCR-ABL1, we could still measure circulating CD26^+^ LSCs while only 7% of patients scored negative for PB LSCs while showing a detectable BCR-ABL1 transcript. When considering the whole cohort of 112 TFR patients, we found an inverse correlation between PB CD26^+^ LSCs number and TFR duration albeit this observation still needs to be validated in a larger series of patients. However, the confirmation that most TFR patients still carry quiescent CML stem cells (molecularly “silent?”) aligns with clinical observation that other factors, likely including a CML-specific immune response, may contribute to CML LSCs control and avoid disease recurrence after TKI discontinuation.

Our data, including a total of 348 patients, furnish for the first time a robust evidence that circulating CML LSCs persist in the majority of CML patients while on TKI treatment or after TKI discontinuation. The clinical value of monitoring residual CD26^+^ LSCs in addition to standard molecular response still needs to be validated; however, our data reinforce the concept that measurement of BCR-ABL1 transcript only may not reflect the actual residual CML LSCs burden. The possibility to easily detect and quantify by flow cytometry PB CML LSCs may open new challenges in CML research and rises novel questions. The number of circulating CD26^+^ LSCs at diagnosis and the slope of their reduction may play a role in achieving an optimal response and subsequently a successful TFR? Is there a LSCs “threshold” that keeps patients from CML relapse? Is CD26^+^ LSCs the target of immune system for disease control? To answer some of these questions, prospective studies evaluating in newly diagnosed CP CML patients, the dynamics of PB CD26^+^ LSCs during different TKI treatment, as well as studies monitoring PB CD26^+^ LSCs prospectively in CML patients that discontinued TKIs, are currently ongoing.

## Ethics Statement

This study was carried out in accordance with the recommendations of Good Clinical Practice. The protocol was approved by the Siena Ethic Committee. All subjects gave written informed consent in accordance with the Declaration of Helsinki.

## Author Contributions

MBo designed and coordinated the study and prepared the manuscript. LA, MD, and IF collected and analyzed data and cowrote the manuscript. AS, SSir, and PPa performed flow cytometry assays and data analysis. LP performed statistical analysis. EA, AI, AGo, SG, BM, PPr, FS, GA, CF, FC, MBr, DC, OM, CB, GC, SSic, AGoz, LL, MC, MA, MT, EU, NS, GR, and AB enrolled patients in the study, collect patient samples and clinical data. DR developed and coordinated flow cytometry assays and cowrote the manuscript. All authors revised and approved the final manuscript.

## Conflict of Interest Statement

EA, MBo, AB, MBr, FC, MC, CF, SG, AI, GR, NS, and MT have received consultancy fees and reimbursement for attending symposia from Novartis, Bristol Mayer Squibb, Pfizer, and Incyte. All the other authors declare no conflicts of interest.
